# Rationale and design of healthy at home for COPD: an integrated remote patient monitoring and virtual pulmonary rehabilitation pilot study

**DOI:** 10.1186/s40814-024-01560-x

**Published:** 2024-10-28

**Authors:** Laurel O’Connor, Stephanie Behar, Seanan Tarrant, Pamela Stamegna, Caitlin Pretz, Biqi Wang, Brandon Savage, Thomas Thomas Scornavacca, Jeanne Shirshac, Tracey Wilkie, Michael Hyder, Adrian Zai, Shaun Toomey, Marie Mullen, Kimberly Fisher, Emil Tigas, Steven Wong, David D. McManus, Eric Alper, Peter K. Lindenauer, Eric Dickson, John Broach, Vik Kheterpal, Apurv Soni

**Affiliations:** 1 Program in Digital Medicine, Department of Medicine, University of Massachusetts Chan Medical School, Worcester, MA 01605 USA; 2 Division of Health System Science, University of Massachusetts Chan Medical School, Worcester, MA USA; 3 CareEvolution, LLC, Ann Arbor, MI USA; 4 Department of Community Medicine and Family Health, University of Massachusetts Chan Medical School, Worcester, MA USA; 5 Office of Clinical Integration, University of Massachusetts Memorial Healthcare, Worcester, MA USA; 6 Division of Health Informatics and Implementation Science, Department of Population and Quantitative Health Sciences, University of Massachusetts Chan Medical School, Worcester, USA; 7 Pulmonary, Allergy, and Critical Care Medicine, Department of Medicine, University of Massachusetts Chan Medical School, Worcester, MA USA; 8 Department of Healthcare Delivery and Population Sciences and Department of Medicine,, University of Massachusetts Chan Medical School–Baystate, Springfield, MA USA

**Keywords:** Chronic obstructive pulmonary disease, Digital phenotype, Mobile integrated health, Digital medicine

## Abstract

Chronic obstructive pulmonary disease (COPD) is a common, costly, and morbid condition. Pulmonary rehabilitation, close monitoring, and early intervention during acute exacerbations of symptoms represent a comprehensive approach to improve outcomes, but the optimal means of delivering these services is uncertain. Logistical, financial, and social barriers to providing healthcare through face-to-face encounters, paired with recent developments in technology, have stimulated interest in exploring alternative models of care. The Healthy at Home study seeks to determine the feasibility of a multimodal, digitally enhanced intervention provided to participants with COPD longitudinally over 6 months. This paper details the recruitment, methods, and analysis plan for the study, which is recruiting 100 participants in its pilot phase. Participants were provided with several integrated services including a smartwatch to track physiological data, a study app to track symptoms and study instruments, access to a mobile integrated health program for acute clinical needs, and a virtual comprehensive pulmonary support service. Participants shared physiologic, demographic, and symptom reports, electronic health records, and claims data with the study team, facilitating a better understanding of their symptoms and potential care needs longitudinally. The Healthy at Home study seeks to develop a comprehensive digital phenotype of COPD by tracking and responding to multiple indices of disease behavior and facilitating early and nuanced responses to changes in participants’ health status. This study is registered at Clinicaltrials.gov (NCT06000696).

**Supplementary Information:**

The online version contains supplementary material available at 10.1186/s40814-024-01560-x.

## Introduction

Chronic obstructive pulmonary disease (COPD) is responsible for 873,000 ED visits and 700,000 hospitalizations each year in the United States, making it a leading cause of morbidity and mortality among Americans [1–3]. This condition, characterized by chronic symptoms such as shortness of breath, coughing, and mucus production, leads to frequent episodes of acute exacerbation [4]. Due to the frequency and severity of exacerbations, one in five COPD patients is readmitted to an inpatient facility within 30 days of hospital discharge [1–3]. Readmissions are often longer in duration and more costly than index admissions [1–3]. This leads to COPD care accounting for a disproportionate share of medical expenditure; the high level of acute care needs attributes to 70% of the $50 billion spent annually on COPD treatment [5–9]. In addition to increasing the cost of care, frequent COPD exacerbations reduce patients’ quality of life and increase their risk of COPD-related mortality [2, 10–12].

Early detection and treatment of acute COPD exacerbation have been shown to improve patient quality of life and decrease the need for emergency services utilization and hospital admission [10]. Furthermore, interventions upstream of an index admission for COPD are more effective than interventions during hospital admission or post-discharge, to prevent further admissions [9]. Put another way, the best way to prevent COPD readmission is to prevent index admission [9]. However, uncertainty remains about the optimal means of predicting and intervening upon impending COPD exacerbations.

Home monitoring systems using symptom reporting and wearable sensors show promise; small studies examining the use of daily symptom telemonitoring have shown that such surveillance facilitates the timely detection of COPD exacerbation [13–15]. However, the burden of daily symptoms and vital sign collection is onerous, and real-world evidence has demonstrated limited adherence to active vital sign collection that requires patient participation. Alternatively, passive collection of biometric data such as oxygen saturation and activity levels minimally adds to patient burden and has been shown to have prognostic value for acute care needs [16]. Further validation of the utility of biometric data signals as clinically actionable alerts for patients and their providers in real-world practice is needed. Additionally, early antibiotic and steroid therapy have been shown to improve both outcomes and patient quality of life, but to initiate treatment, clinicians must be alerted to clinical deterioration and be empowered to intervene promptly [10, 17]. Therefore, in tandem with the optimization of monitoring strategies, a systematic approach to review and respond to alerts based on consolidated data streams is needed to prevent added burden for the patients and their medical team. The present study has been designed to leverage ongoing efforts to configure a multimodal virtual care intervention for COPD patients and evaluate the feasibility of its implementation. Empowering patients with tools to help them expedite the recognition of worsening symptoms and removing barriers to care access are two critical components of improving clinical outcomes and patient quality of life.

The objective of the current study is to conduct a feasibility trial of the “Healthy at Home” program, a multimodal intervention aimed at preventing emergency services utilization and hospitalization among patients living with COPD. The program features a digital platform linked to a wearable health-monitoring device, virtual pulmonary support to facilitate complex care management, and access to an around-the-clock mobile integrated health (MIH) service that provides on-demand, in-home visits for evaluation and treatment of acute symptoms.

## Methods

### Setting and participants

This study was performed at an urban, academic tertiary care center. Inclusion criteria for participation included receiving healthcare through the hospital system of interest, being 18 years of age or older at the time of recruitment, and carrying a diagnosis of COPD. Subjects were also required to have access to a smartphone (iPhone or Android) with internet access to download and use the study apps and to live within the regional geographic area served by the system’s MIH program. Those who lacked the capacity to consent, did not understand English, did not have internet access, were enrolled in another investigational clinical trial at the time of enrollment, or were (or had previously been) enrolled in any Wellinks pulmonary support program were excluded from the study. The study was approved by the WIRB-Copernicus Group Institutional Review Board and is registered at Clinicaltrials.gov (NCT06000696).

### Recruitment

Screening for participant eligibility was performed via a query of the system’s electronic health record (EHR) for patients with a diagnosis of COPD. The number of ED visits and hospitalizations, along with the number of COPD-related medication changes in the 2 years before study enrollment, were considered proxy variables for COPD severity and treated as a count variable. We refined our recruitment to those patients that were within the second through fourth quintile of the count variable such that our cohort was comprised of patients with moderate-severity COPD. On a per-case basis, exceptions to this strategy were accommodated when patients were directly referred to the research team by the clinical team.

**Fig. 1:**
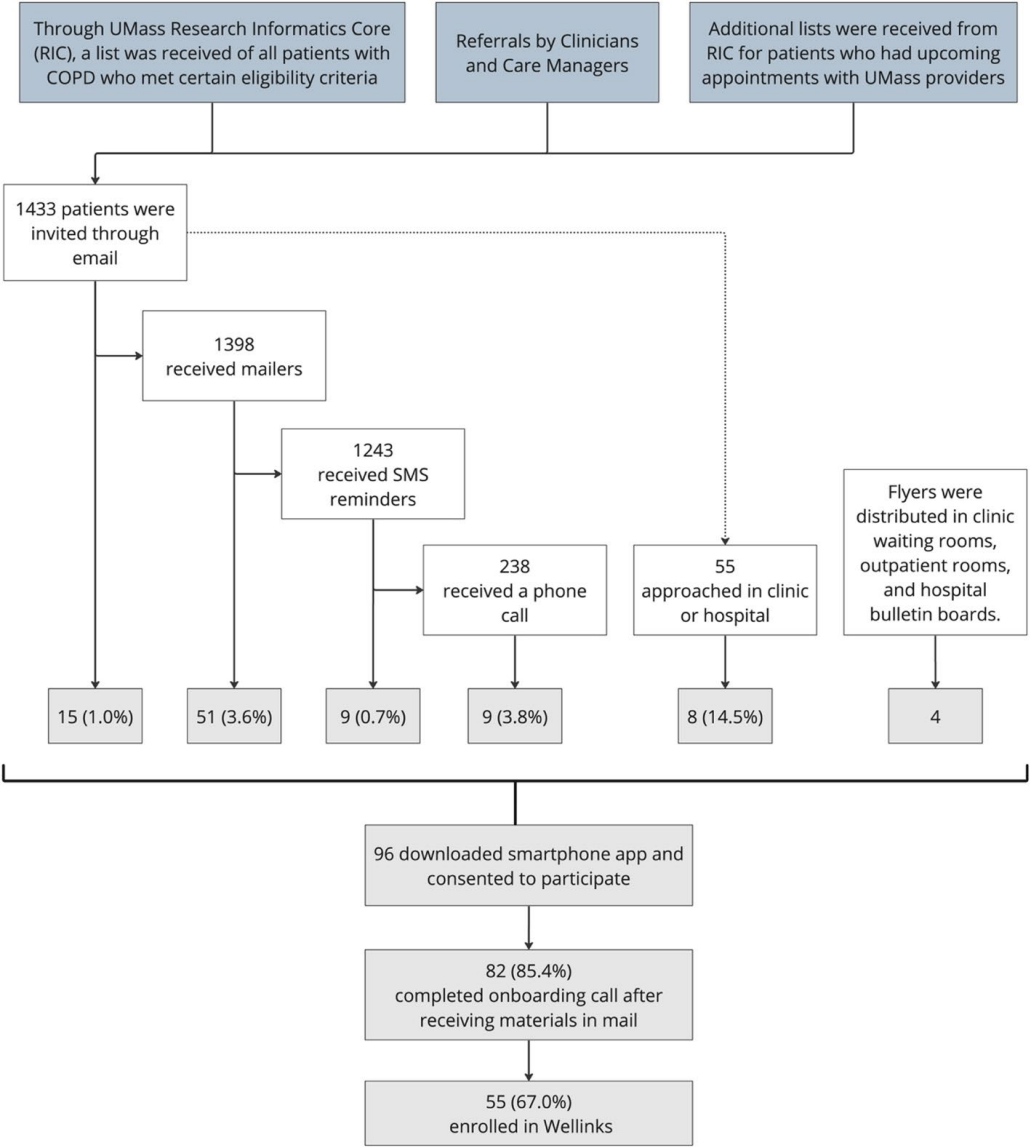
Participant recruitment workflow: Potential participants were primarily identified via a query of the institutional electronic health record and invited via email, followed by paper mailers and SMS messages. Potential participants who were hospitalized or attending ambulatory appointments were approached by the study team in person or self-identified themselves from flyers advertising the study

All participants were initially invited via email. Non-response to email solicitation was followed by two text messages within 2 weeks of the email invitation and a paper mailer sent to prospective participants’ homes. Patients who were identified as prospective participants and who were hospitalized or had upcoming ambulatory pulmonary clinic appointments were approached in person by the study team at the medical center. Finally, flyers advertising the study were placed in high-visibility places such as clinic waiting rooms. The recruitment strategy and number of participants approached are summarized in Fig. 1. Invitations for recruitment were conducted in waves ranging from 500 to 3000 patients based on the composition of the study cohort after each cycle to achieve a balanced representation of risk categories and sociodemographic factors.

### Procedure

Participants who expressed interest in participating were given instructions to download the study app, a custom interface created through the MyDataHelps platform (Supplemental File 1). MyDataHelps is a mobile application created by CareEvolution to give a platform for researchers and participants to engage in clinical research. Through this study app, participants completed eligibility questions to ensure that they met all inclusion criteria for the study. If they were determined to be eligible, participants were subsequently prompted to digitally review and sign an informed consent form through the study app. Because eligibility screening and enrollment could be performed through the app, participants could enroll entirely remotely.

**Fig. 2:**
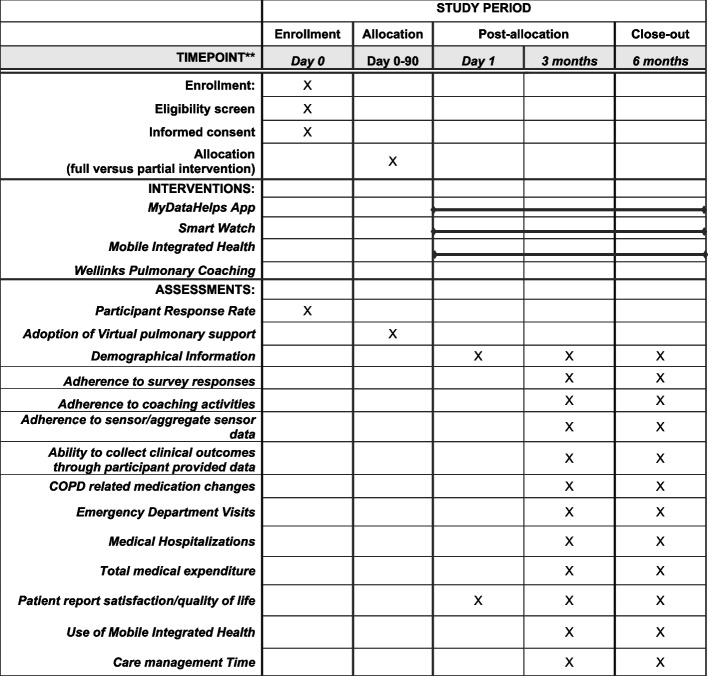
SPIRIT depictionof study activity and outcomes

Once participants completed consenting procedures, a welcome kit was shipped to their residence containing all necessary study-related materials including a Fitbit Charge 5 smartwatch and additional literature about the study and the affiliate MIH program. Subjects were then prompted to schedule an onboarding call with a member of the study team where they were guided through setting up and using the smartwatch, educated on the use of the study app, and provided any additional support needed to initiate participation in the study. Once onboarded, participants were asked to partake in study procedures for 6 months. A summary of all components of the study and their interconnectedness is depicted in Table 1. A Standard Protocol Items: Recommendations for Intervention Trials (SPIRIT) Figure for the study is depicted in Fig. 2.

**Table 1:** Healthy at home intervention components: This multimodal intervention included a study app, smartwatch, access to acute care through a mobile integrated health program and virtual pulmonary support services

Intervention component	Description	Interaction with other components	All participants or opt-in
MyDataHelps App (main study app)	Houses all participant-facing forms and questionnaires including screening and consent. Prompts participants to complete scheduled assessments. Receives information from biosensors and is linked to participants’ EHR and claims data to aggregate all data streams. Generates momentary assessments triggered by participant responses or biometric data	All participant-level MyDataHelps data is visible to the Wellinks and MIH team on the study dashboard. Clinical data can trigger alert to participants through the app suggesting a home assessment from the MIH team and connect participants directly with the MIH visit request line	All
Fitbit Smartwatch	Collects biometric data. All data is visible to participants, study team, MIH clinicians, and Wellinks coaches through the MyDataHelps	Biometric patterns trigger alert to patients suggesting MIH visits. Biometric data is visible to clinical and research teams	All
Mobile Integrated Health Program (community paramedics and MIH physicians)	Community paramedics are available on-demand to perform home visits to evaluate and treat participants experiencing acute symptoms with support from a supervising physician. The program is specifically equipped to initiate treatment for COPD exacerbation	MIH team can view participant-level data in MyDataHelps. MIH visits offered to participants reporting worsening symptoms or exhibiting concerning biometric data through the study app. Research and Wellinks team refer participants to MIH team for all acute clinical concerns	All
Wellinks Virtual Pulmonary Rehabilitation Program	Coaching program to support participant education, treatment adherence, and goal setting, as well as a virtual pulmonary rehabilitation. Hosted on a separate program-specific app	Wellinks team can view participant-level data in MyDataHelps to support coaching plans. Wellinks can contact MIH team with any acute concerns requiring clinical evaluation	Opt-In
Wellinks equipment kit (spirometer, pulse oximeter, exercise equipment	Used with the Wellinks team to support pulmonary coaching plan and collect additional relevant biometric data	Spirometry and pulse oximetry data is integrated with the MyDataHelps app and is visible to the study and clinical teams	Opt-In

### Participant reported outcomes

Study participants were asked to complete a series of instruments throughout the 6-month study period through the MyDataHelps app. Participants were notified of outstanding surveys and prompted to complete them through app push notifications according to the study schedule. In addition to demographic questions asked at baseline, participants were prompted to complete surveys including the NIH-PROMIS COPD questionnaires, Patient Activation Measure, Modified Medical Research Council Dyspnea Scale, and a patient satisfaction measure at enrollment, 3 months, and 6 months (Table 2) [18–21]. Participants were also prompted to complete the COPD Assessment Test monthly and a single-item wellness measure weekly [22]. Momentary assessments for participants were assessed using CLEAR-Sx, Ex, and Rx tools; these were triggered based on participants reporting not feeling well on the single-item wellness measure or by wearable device data (Table 3) [16, 23].

**Table 2:** Participant-facing assessments: Participants were prompted to complete all assessments through the Care Evolution Healthy At Home study app. The app prompted participants to complete each assessment as they were due and sent reminders for delinquent assessments

Questionnaire	Frequency
Baseline	Once (available upon enrollment)
COPD Assessment Test	Monthly
NIH-PROMIS COPD	0, 3, and 6 months
CLEAR-Sx, Ex, and Rx	Ad hoc (administered when metrics such as oxygen saturation, spirometry data [if available], activity indices [if available] indicate a potential COPD exacerbation)
Patient Activation Measure	0, 3, and 6 months
mmRC (Modified Medical Research Council) Dyspnea Scale	0, 3, and 6 months
Patient Satisfaction	0, 3, and 6 months
Single-item wellness	Weekly
Exit Survey	6 months

**Table 3:** Clinical alert triggers. The clinical alert metrics triggered app notifications to prompt participants to request a medical evaluation if they were feeling unwell. The app enabled participants to quickly call the mobile integrated team for an on-demand acute home visit with a “one-touch” option to dial the MIH hotline phone number

Metric	Definition for flag in dashboard
SpO2 (triggers CLEAR-Sx, Ex, Rx survey)	Fall in SpO2 below 90% for 2 consecutive days or a fall of 4% or higher below 7-day rolling average for 2 consecutive days
FEV1, FVC, PEF, and IC (triggers CLEAR-Sx, Ex, Rx survey)	Fall of 1.65 SD or greater below 7-day rolling average for 2 consecutive days
Quality of Activity (triggers CLEAR-Sx, Ex, Rx survey)	Calculated from Activity Index and Regularity Index. Fall of 1.65 SD or greater below 7-day rolling average for 2 consecutive days
Exacerbation (categorized by answers to CLEAR Sx, Ex, and Rx survey)	Anthonisen criteria (Category I, II, or III) based on self-report of symptom data

### Remote patient activity monitoring

Participants were asked to wear the smartwatch daily, including at night, to collect data including daily steps, heart rate, oxygen saturation, and sleep patterns. This information was visible to study coordinators and investigators, as well as MIH paramedics, through the MyDataHelps platform. The data were used to develop automated prompts for participants to provide momentary assessments using CLEAR surveys.

### Virtual comprehensive pulmonary support service

All participants were presented with the option to enroll in a virtual pulmonary care program that provides multimodal support for COPD patients, through a company called Wellinks. The option to enroll in this was made available through the MyDataHelps during the first 90 days of study participation. The program includes coaching to support participant education, treatment adherence, and goal setting, and a virtual pulmonary rehabilitation program that provides a home-based exercise plan including safety instructions and COPD-specific breathing techniques. Additionally, Wellinks participants received a second equipment kit containing a spirometer, pulse oximeter, ten-foot tape measure, exercise band, and an exercise guide. The virtual pulmonary support program team coordinated introductory calls with participants to download their app and configure the spirometer and pulse oximeter devices via Bluetooth which are used to collect additional biometric data. After a comprehensive personalized assessment, a schedule for regular sessions with a health coach was created for participants. All coaching was scheduled and joined through the program’s app, with board-certified health and wellness coaches or nurse practitioners.

In the event of an acute clinical concern, the Wellinks team’s protocol was to instruct participants to contact the MIH clinician or their healthcare provider. The Wellinks team was also empowered to contact the on-duty MIH clinician directly. Participants who enrolled in the Wellinks program were classified as the “full-intervention” group, and those who enrolled in the study but opted not to participate in the Wellinks program were considered the “partial-intervention” group.

### MIH integration

To provide on-demand, field-based clinical support to participants, all enrolled participants were invited to utilize the institution’s affiliate MIH program for the duration of their participation [24]. MIH programs are new models of care that leverage mobile resources, including specially trained paramedic-level clinicians, called community paramedics, to evaluate and care for patients in an out‐of‐hospital environment in coordination with health systems [24, 25]. By providing field-based care, such programs are intended to eliminate barriers that patients encounter in accessing timely management of acute illness. They complement digital and remote clinical interventions by providing flexible, face-to-face evaluation and treatment to patients. During episodes of acute clinical symptoms (such as worsening shortness of breath), participants, their caregivers, and the Wellinks team were empowered to request an MIH visit. The community paramedics are available 24 h a day, 7 days a week, and present to participants’ homes within 2 h of a request. Upon arrival, they evaluate and treat participants, aided by mobile diagnostic tools and medications as well as telehealth support from an on-call supervising physician, then develop a plan of care with the participant, which usually entails treatment in the home and intensive care coordination with the participant’s ambulatory providers. The MIH program is specifically equipped to initiate treatment for COPD exacerbation with inhaled bronchodilators and parenteral antibiotics and steroids. If a participant is too acutely ill to remain at home, the participant is diverted into the emergency services system. To streamline care, the community paramedic team and their supervising physicians had access to participants’ study dashboards so that they could review their aggregated clinical data and recent changes in biometric patterns.

In addition to the education and literature provided during study onboarding, the participant-facing MyDataHelps app contained a “Call MIH” button which enabled the participant to easily contact MIH for an in-home assessment if they felt unwell by directly connecting them with the MIH request line. The MyDataHelps app was set to issue a prompt to the participant to consider calling MIH when pre-set metrics are consistent with a potential COPD exacerbation (Table 3). Finally, research coordinators were instructed to refer participants to the MIH hotline if any clinical concerns were raised while on a phone call with participants. The MIH program did not proactively provide care because of alerts or flags observed from participant-reported data or biometric signals in this pilot phase. Clinical care provided by the MIH paramedics for the study’s participants was identical in practice to the care provided to patients outside the study.

## Analysis plan

**Fig. 3:**
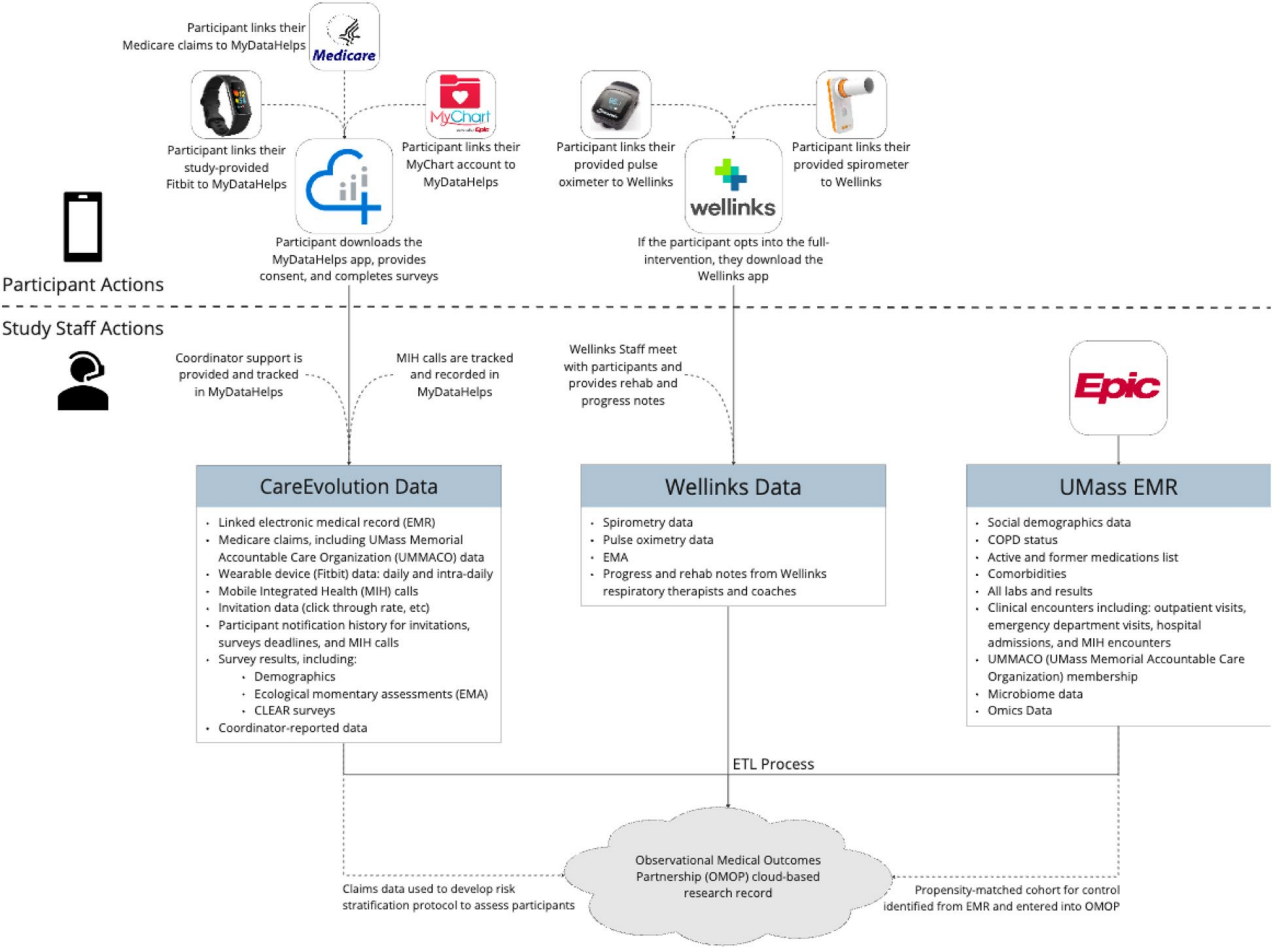
Participant data flow: Data linked to each participant was collected from several sources throughout the study period including participant-entered assessments, Fitbit and spirometer-obtained biometric data, the Epic Electronic Health Record, Wellinks, and Medicare claims data

Participants are followed longitudinally for 6 months in the pilot study, during which measures of feasibility, clinical efficacy, and operational efficacy will be examined. The feasibility measures observed in the two intervention arms include the response rate for study participants, the adoption rate for virtual pulmonary support, adherence for survey and sensor data, adherence to coaching activities, ability to aggregate sensor data, and ability to measure clinical outcomes through participant-provided data. Table 4 describes planned feasibility outcomes, how they will be operationalized, and the target thresholds indicating feasibility for a larger trial. Figure 3 outlines all data collected during the study.

**Table 4:** Feasibility outcomes: A summary of chosen study feasibility outcomes, operationalization of measurement and target thresholds for feasibility

Conceptual outcome	Operational measure	Feasibility benchmark
Recruitment	Rate of enrollment; Number of participants approached, met eligibility and enrolled	• Enrollment of 100 subjects in 12-month study period • Enroll ≥ 20% of invited participants
Retention rate	Number of enrolled participants who remain in the study 6 months	• Participant Retention ≥ 70%
Adoption of virtual pulmonary rehabilitation	Number of participants who opt in to Wellinks	• Participant adoption ≥ 50%
Wearable monitor utilization	Amount of time that participants transmit detectable biometric signals from Fitbit	• Median daily watch use ≥ 17 h • Median watch use ≥ 126 days • Median daily heart rate detection ≥ 1008 min
Completion of study instruments	Number of participants that complete each study instrument	• ≥ 80% of all required study instruments completed by participants
Data abstraction completion	Completeness of required participant clinical data from health records	• ≥ 90% completion of participant clinical data fields

### All feasibility outcomes will be reported descriptively

Clinical and operational efficacy will be compared across all three groups of COPD participants: full-intervention, partial-intervention, and a control group (no intervention) identified from the EMR using a novel synthetic control study strategy that has been endorsed by the FDA for generating real-world evidence [26, 27]. The clinical efficacy measures include medication requirements, healthcare utilization related to acute or urgent care, total medical expenditure, and, in the intervention arms, participant-reported outcomes (COPD severity, COPD exacerbations, patient activation measure, patient satisfaction, modified Medical Research Council Dyspnea Scale). Lastly, operational efficacy includes time spent during MIH visits, MIH visit escalation to acute care rate, and care management time.

Descriptive analysis will be used to characterize the feasibility outcomes. Continuous measures will be described using mean or median depending on the underlying distribution, and categorical variables will be described as proportions. Associations with sociodemographic characteristics and GOLD classification of COPD severity will be assessed for feasibility outcomes. Count models (Poisson, negative binomial, or zero-inflated negative binomial) will be used to compare healthcare utilization over the course of the study. Continuous models will be used to compare medical expenditure. GEE models with logit or linear link will be used to assess changes over time for COPD severity measurements. Table 5 depicts planned clinical and operational outcomes, operationalization of measurements, and preliminary analysis plans for each outcome.

**Table 5:** Clinical and operational outcomes: A summary of chosen clinical outcomes, operationalization of measurement, analysis plan

Conceptual outcome	Operational measure	Analysis plan
Participant perceived COPD severity	• Patient Activation Measure • (Modified Medical Research Council) Dyspnea Scale	All instrument scores will be treated as continuous variables. Regression will be performed using GEE models with logit or linear link. Scores will be compared from participants at baseline and 3 and 6 months
Patient quality of life	• COPD Assessment Test • NIH-PROMIS COPD • Single Item Wellness	
Patient satisfaction	• Patient Satisfaction Measure • Study Exit Survey	
COPD-related medication changes	• Number of COPD-related medication changes during study period	Outcomes will be treated as count variables. Count models (Poisson, negative binomial, or zero-inflated negative binomial) will compare outcomes between the full-intervention, partial-intervention, and synthetic control
ED utilization	• Number of ED visits during study period	
Hospitalization	• Number of COPD-related hospitalizations during study period	
Medical expenditures	• Total claims costs during study period	Claims will be considered in aggregate for all costs paid during the study period; they will be treated as a continuous variable. Continuous models will compare outcomes between the full-intervention, partial-intervention, and synthetic control
COPD-related MIH effort	• Number of MIH visits completed • Median MIH Visit time • Median physician effort time per MIH visit	Descriptive

## Discussion

The Healthy at Home study addresses a need to remove barriers to preventative and rehabilitative care for COPD patients and recognize potential indicators of COPD exacerbations. The current mainstay of COPD care is centered around acute disease management and services in post-acute settings. However, there is an unmet need for providing proactive care to avert or diffuse an acute exacerbation before hospitalization occurs. Prior studies have focused on remote patient monitoring to detect evidence of acute exacerbation, but there is a dearth of knowledge about the operational impact and implementation of these interventions. There is also limited data on whether such monitoring initiatives are cost-effective [15]. This study aims to demonstrate the feasibility of conducting a larger-scale study using the novel synthetic control design to generate real-world evidence for clinical, operational, and cost outcomes related to the provision of the “Healthy at Home” program.

By employing the use of a Fitbit device, on-demand acute in-home healthcare visits, and, for full-intervention participants, a pulse oximeter, spirometer, and virtual health coaching, participants will have a greater ability to view and act on their personal health metrics. In addition to monitoring their own health and being encouraged to contact the MIH program for health-related concerns, participants’ health and survey data will be used to further understand the most important factors predictive of COPD-related exacerbations. The use of integrated data steams combining participant-reported information, biometric signals, and EHR patterns will generate a rich multifaceted digital phenotype of the COPD disease process which can be used to predict future clinical outcomes and facilitate proactive intervention when deterioration is predicted.

We also expect that this important addition to the knowledge base of COPD care will come with a limited burden to participants, as the majority of the data collected is passive. It is expected that participants will spend about 10 min per week on study activities, with an additional hour per week of pulmonary coaching and exercises for those enrolled in the full-intervention portion of the study. We predict that this consciously low physical and cognitive burden to participants will result in excellent implementation outcomes such as high retention, fidelity to the study protocol, and adoption of the interventions including the use of the MIH program.

The current study is one of many programs implemented in recent years attempting to reduce COPD-related readmissions. The Centers for Medicaid and Medicare Services has listed COPD as a target condition for the Hospital Readmission Reduction Program (HRRP). This program imposes penalties on health systems in relation to their readmission rates in an effort to promote innovation and reduce 30-day readmission. Experts have raised concerns that the punitive measures associated with the HRRP could lead to efforts focused on reducing readmissions without equal consideration for improvement in the quality of patient care [28–32]. Existing literature has focused on the root causes of readmissions, identifying areas in which improvement is needed [3]. Evidence surrounding COPD readmissions shows that multimodal interventions that include frequent assessment, care management, and pulmonary coaching or rehabilitation have the highest efficacy for preventing readmission [9].

It is expected that this study will have implications for the standard of care provided to patients at risk of admission for COPD exacerbations. If successful, increased implementation of at-home monitoring and virtual pulmonary support services may be established for patients who would benefit from this level of care. Additionally, this information can be shared with healthcare providers, such as community paramedics to assist them in providing a more proactive and robust level of care for their patients. It is also a goal of the study that these activities will help to decrease the cost acquired for patients who accumulate high levels of healthcare spending due to repeated hospitalizations and acute care needs. As a feasibility study, the current project aims to establish the impacts of study activities and expand both the population and the research partners in future analysis of this population.

By enrolling all-comers, agnostic to insurance type, residing in the towns serviced by the affiliate MIH program, it is expected that we will enroll a diverse population in reference to race, ethnicity, and socioeconomic status. However, this study is limited to those who have established health care within the system of interest and those who reside in the fairly urban areas surrounding these hospitals. Additionally, those who do not speak English are excluded from this study. Future studies should focus on a more diverse population based on geographical location and language, as there are barriers to care in those populations that will not be seen in the current study. Additionally, participants are required to have a smartphone with the ability to access the internet at home to enroll on the study app, potentially excluding a subset of the population who do not have these resources.

The Healthy at Home program’s feasibility study aims to create a foundation around which future, more expansive studies can be constructed. Independent or ancillary studies could be designed, possibly in coordination with pre-existing studies in digital medicine, to allow this novel multimodal platform to be harnessed by those aiming to better understand other disease states and populations. In the case of studying other disease states, the structure of Healthy at Home could easily be modified to work with other healthcare vendors who may be specialized to care for a specific population’s needs. Past simple expansion of study protocols, the information gathered from studies built on the foundation of Healthy at Home may be used in the long-term to grow patient care using this digital, multimodal approach. It is the hope to enhance both study protocols for future investigations and medical care for patients in a multi-phasic approach, by which optimizations are made over a period of time, building upon each other. Using biometric data gathered from participants, prognostic digital biomarkers may be able to be used to help patients understand when they may be at a heightened risk of needing acute care. This preventative care may also be used in coordination with a virtual, digital coach, enabling patients to be better notified of when they may be at risk or when their vital signs are outside a specified range and to seek interventive care early in the course of a threatened exacerbation. These optimizations, as well as others yet-to-be-articulated, may eventually aid in the elevation of the standard of care provided in both research and practice.

## Conclusions

The Healthy at Home study’s long-term goals are to develop new tools and modes of care to better treat and understand the needs of patients with COPD. This is done by determining the feasibility of this type of digital study, as well as examining the efficacy of the multimodal interventions, across both the partial- and full-intervention groups. Furthermore, we hope to use the data gathered from this current study to inform an improved digital dashboard for use by the MIH clinical team to provide them with a more comprehensive, longitudinal history of their patients’ health. This multimodal approach may aid patients to better manage their COPD symptoms by more closely monitoring their health and providing them with multiple avenues to prevent acute care needs, including through the ability to call community-based medical providers and through regular use of the virtual pulmonary support coaching.

## Supplementary Information

Additional file 1. Patient-facing app through care evolution.

## Data Availability

The protocols and datasets used during the current study are available from the corresponding author upon reasonable request.

## Abbreviations

COPD: Chronic obstructive pulmonary disease
MIH: Mobile Integrated Health
EHR: Electronic health record
HRRP: Hospital Readmission Reduction Program
