# Supplementary material for: Rationale and design of healthy at home for COPD: an integrated remote patient monitoring and virtual pulmonary rehabilitation pilot study
Source: Pilot Feasibility Stud. 2024 Oct 28;10:131. doi: 10.1186/s40814-024-01560-x (PMC11520050; doi:10.1186/s40814-024-01560-x)
Supplement: Supplementary file 1 — Additional file 1. Patient-facing app through care evolution. [file 40814_2024_1560_MOESM1_ESM.docx]

**Supplement 1:** Patient-Facing App through Care Evolution. The Healthy at Home App facilitates participant recruitment and consent, participant-facing data collection, and participant engagement with the multimodal components of the study.

*Taken on an iPhone SE running iOS 15.5*


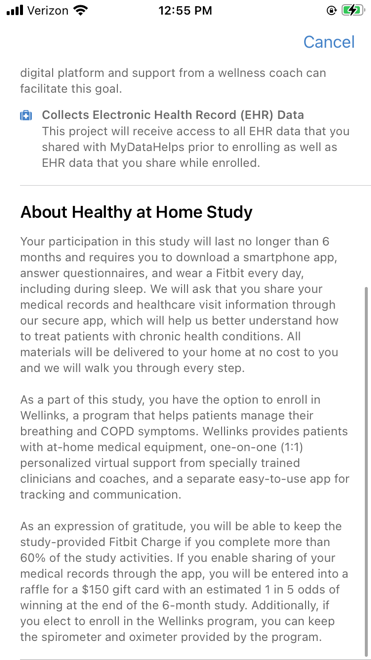

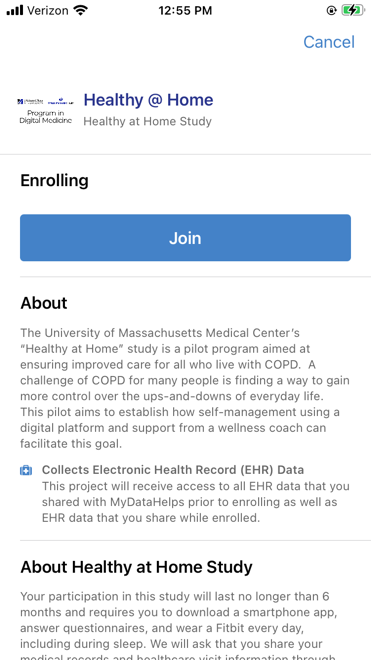


Join study page


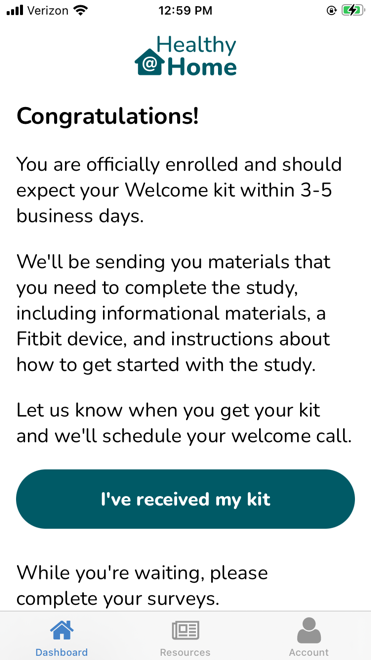

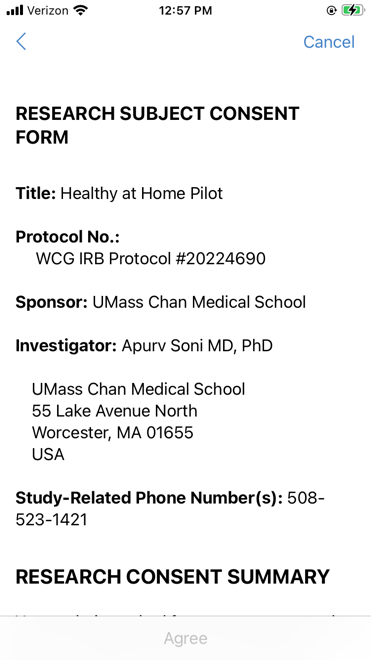

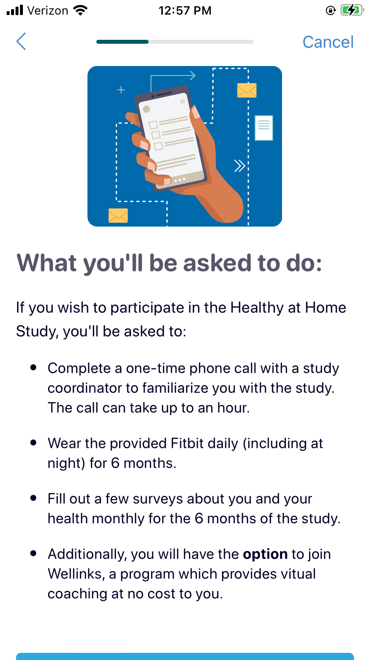

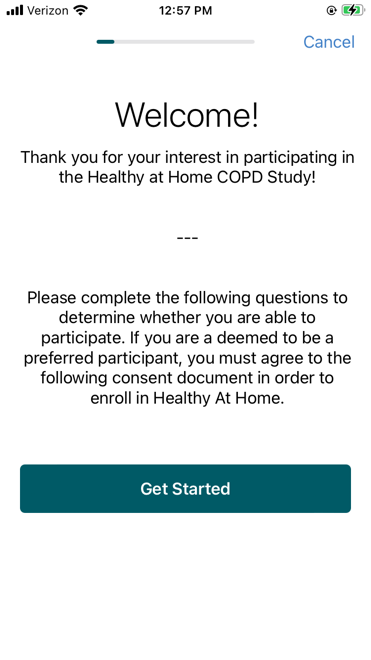


Study app home page, stage 1

Study Consent form

Welcome Pages


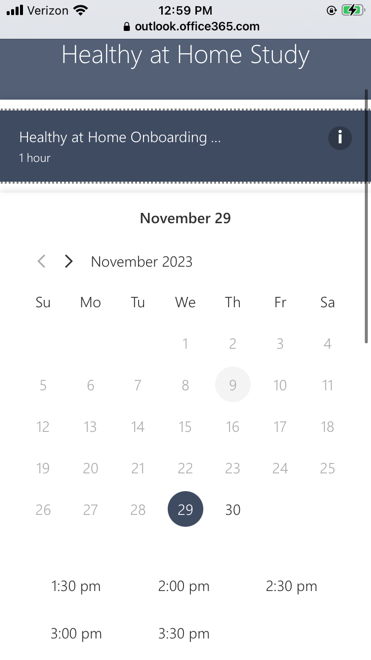

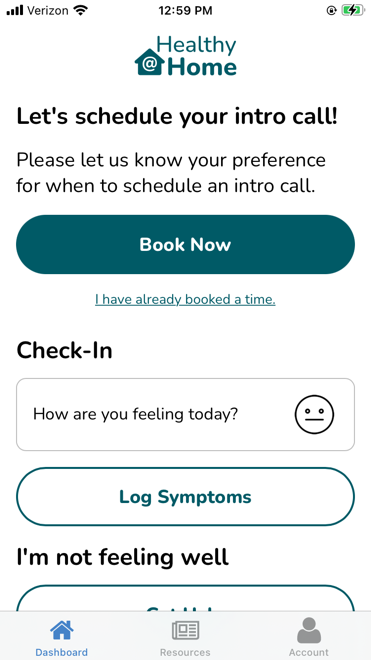

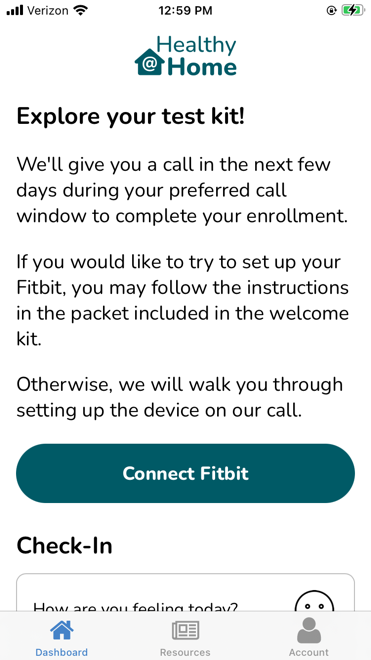


Microsoft Bookings scheduling page

Study app home page, stage 3

Study app home page, stage 2


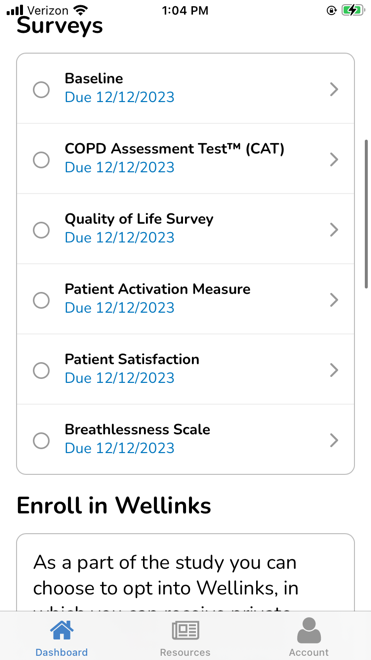

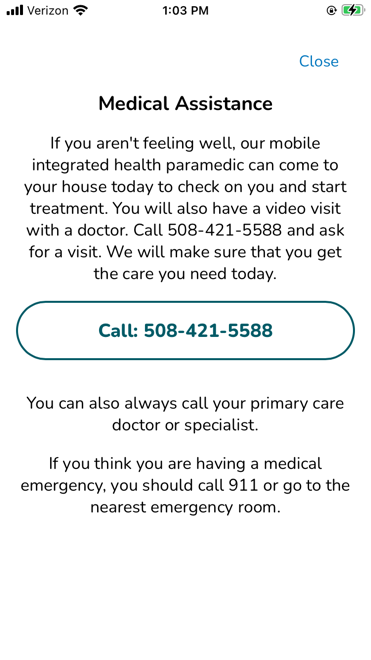

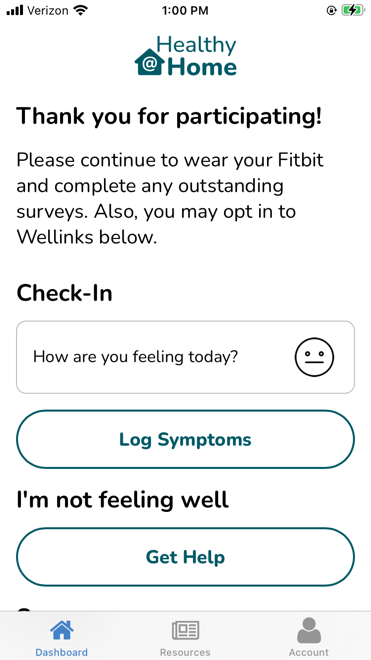

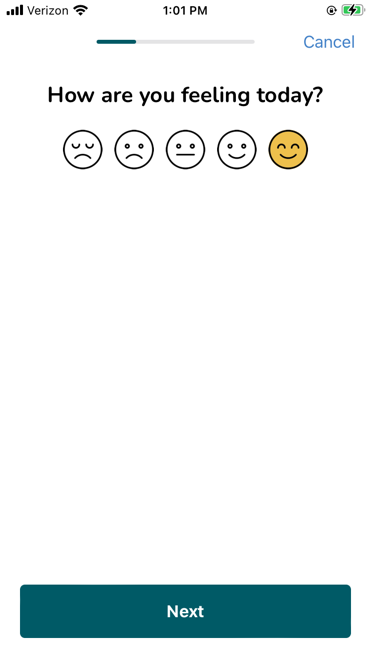


Survey tasks

Call MIH “get help” page

Study app home page after setup

Daily check in page


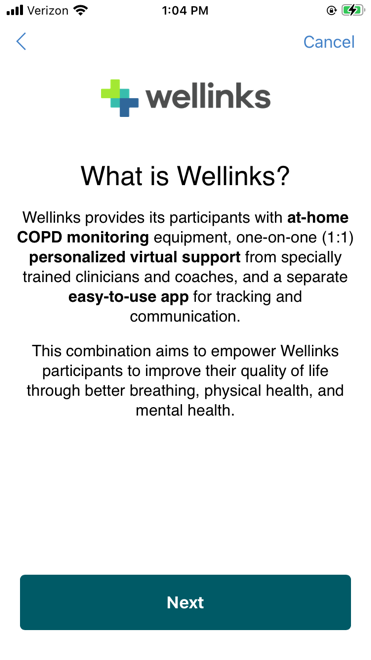

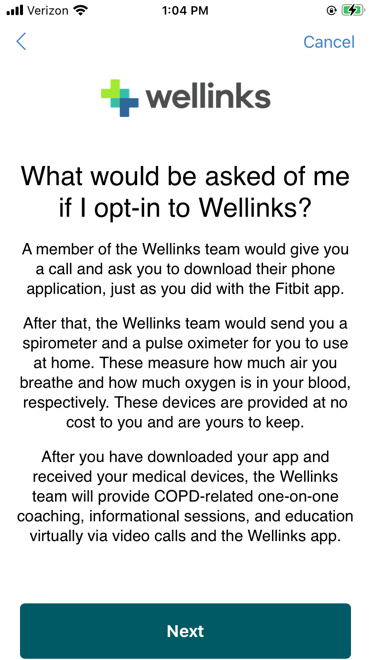

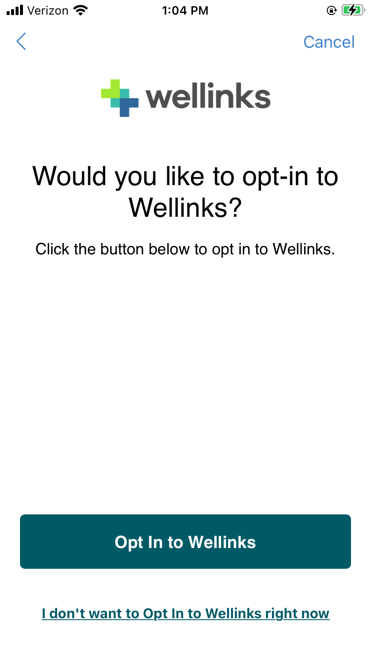

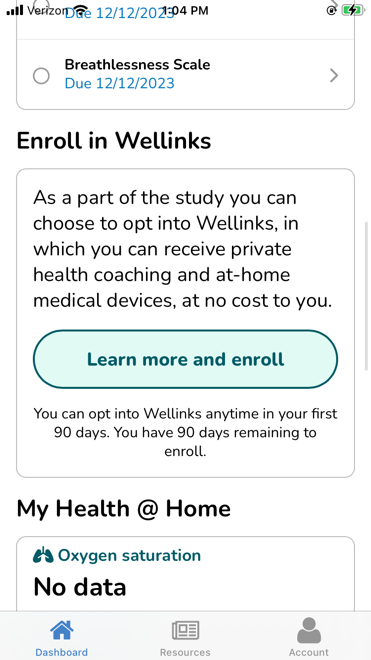


Wellinks opt-in 3

Wellinks opt-in 2

Wellinks opt-in 1

“Enroll in Wellinks” button


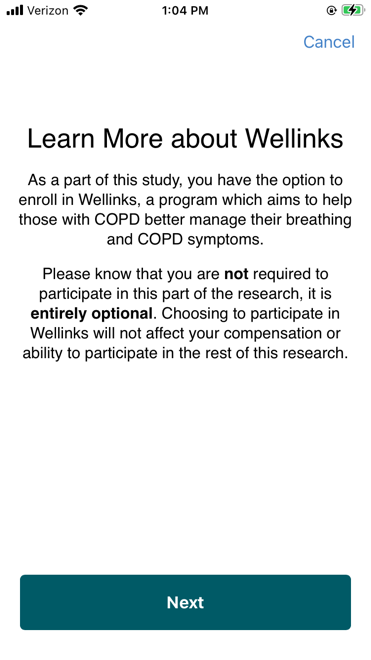

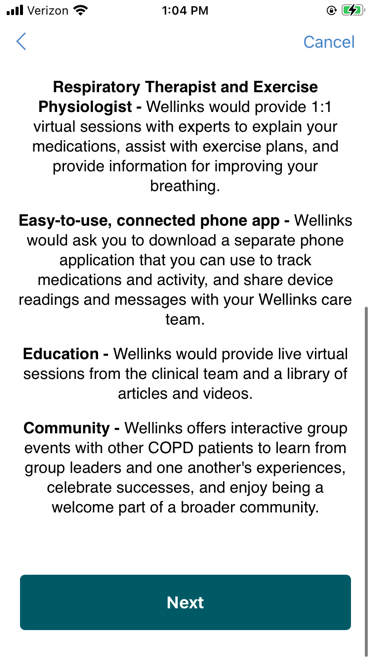

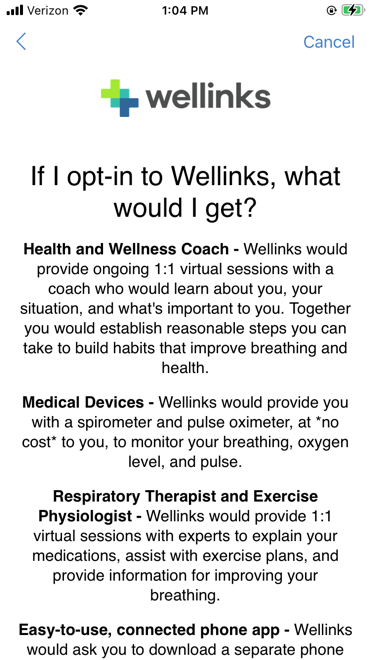


Wellinks opt-in 6

Wellinks opt-in 5

Wellinks opt-in 4


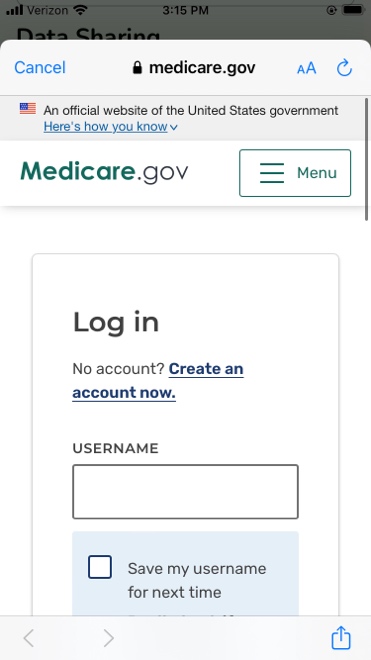

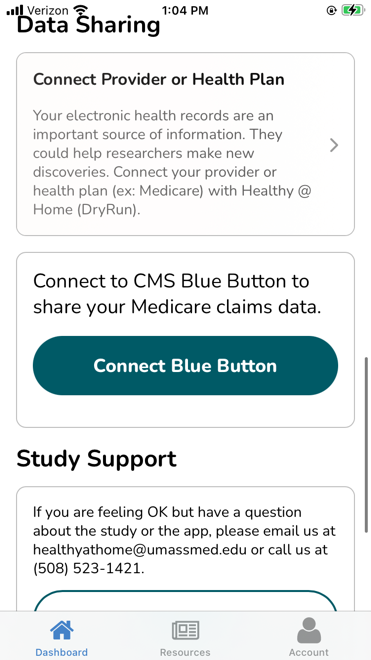


CMS Claims data sharing login

Study app data sharing options
